# Marked QTc Interval Prolongation Associated With Dronedarone in Paroxysmal Atrial Fibrillation and No Structural Disease

**DOI:** 10.7759/cureus.99543

**Published:** 2025-12-18

**Authors:** Sebastian Hernandez Mejia, Joud Fahed, Rayna Isber, Nidal Isber

**Affiliations:** 1 Internal Medicine/Cardiology, Richmond University Medical Center, New York, USA; 2 Internal Medicine, Ascension Saint Agnes Medical Center, Baltimore, USA; 3 Biology, Barnard College, Columbia University, New York, USA; 4 Electrophysiology, Richmond University Medical Center, New York, USA

**Keywords:** antiarrhythmic agents, athena trial, dronaderone, pallas trial, qt interval prolongation

## Abstract

Dronedarone, a multichannel antiarrhythmic drug, was developed as a safer alternative to amiodarone for atrial fibrillation. Although initially considered to have a lower proarrhythmic risk, post-marketing data and clinical experience suggest otherwise. We describe the case of a 44-year-old male with paroxysmal atrial fibrillation and no structural heart disease who developed marked QTc prolongation while receiving dronedarone, despite being asymptomatic. This case highlights the potential for clinically silent but significant QTc prolongation during dronedarone therapy, underscoring the importance of careful ECG monitoring, even in low-risk patients.

## Introduction

QT interval prolongation is a well-recognized risk factor for potentially life-threatening arrhythmias, including torsades de pointes, and can be precipitated by a variety of medications, including antiarrhythmic agents. Dronedarone, a structural analogue of amiodarone, is generally considered to have a more favorable safety profile, particularly regarding long-term organ toxicity [1,2]. However, despite its perceived safety, dronedarone has been associated with rare but clinically significant prolongation of the QT interval, which may predispose susceptible patients to arrhythmic events [3,4].

We present the case of a patient who developed notable QT prolongation after initiation of dronedarone therapy, as documented on serial ECGs. This case underscores the importance of careful ECG monitoring when prescribing dronedarone, even in patients without known risk factors for QT prolongation. By highlighting this case, we aim to raise awareness among clinicians about this rare adverse effect and discuss its implications for clinical practice.

## Case presentation

A 44-year-old physically active male (body mass index: 25 kg/m²) with a two-year history of symptomatic paroxysmal atrial fibrillation was referred for evaluation of recurrent episodes requiring repeated electrical cardioversion. He had no history of hypertension, thyroid disease, or structural heart disease. Baseline laboratory testing revealed normal renal function (estimated glomerular filtration rate: >90 mL/minute/1.73 m²), normal liver enzymes, and normal thyroid function. The patient had mild, untreated obstructive sleep apnea, a known contributor to atrial fibrillation recurrence, though he had declined initiation of continuous positive airway pressure therapy.

Comprehensive cardiac evaluation was unremarkable. A transesophageal echocardiogram performed during cardioversion showed normal cardiac structure, no valvular disease, no regional wall-motion abnormalities, and a preserved ejection fraction of 60%. A prior transthoracic echocardiogram demonstrated similarly normal findings. A nuclear stress test showed no ischemia. There was no family history of sudden cardiac death. He reported excellent functional capacity with no exertional limitations and no symptoms of heart failure.

Ambulatory cardiac monitoring (CardioNet) demonstrated persistent atrial fibrillation with intermittent rapid ventricular response. Prior rhythm-control therapy included propafenone 325 mg twice daily for several months, along with apixaban 5 mg twice daily. He was also taking pantoprazole, which has minimal cytochrome P450 interaction and does not affect propafenone metabolism. Despite adherence, he continued to experience recurrent atrial fibrillation requiring three synchronized cardioversions, and propafenone was therefore discontinued.

**Figure 1:**
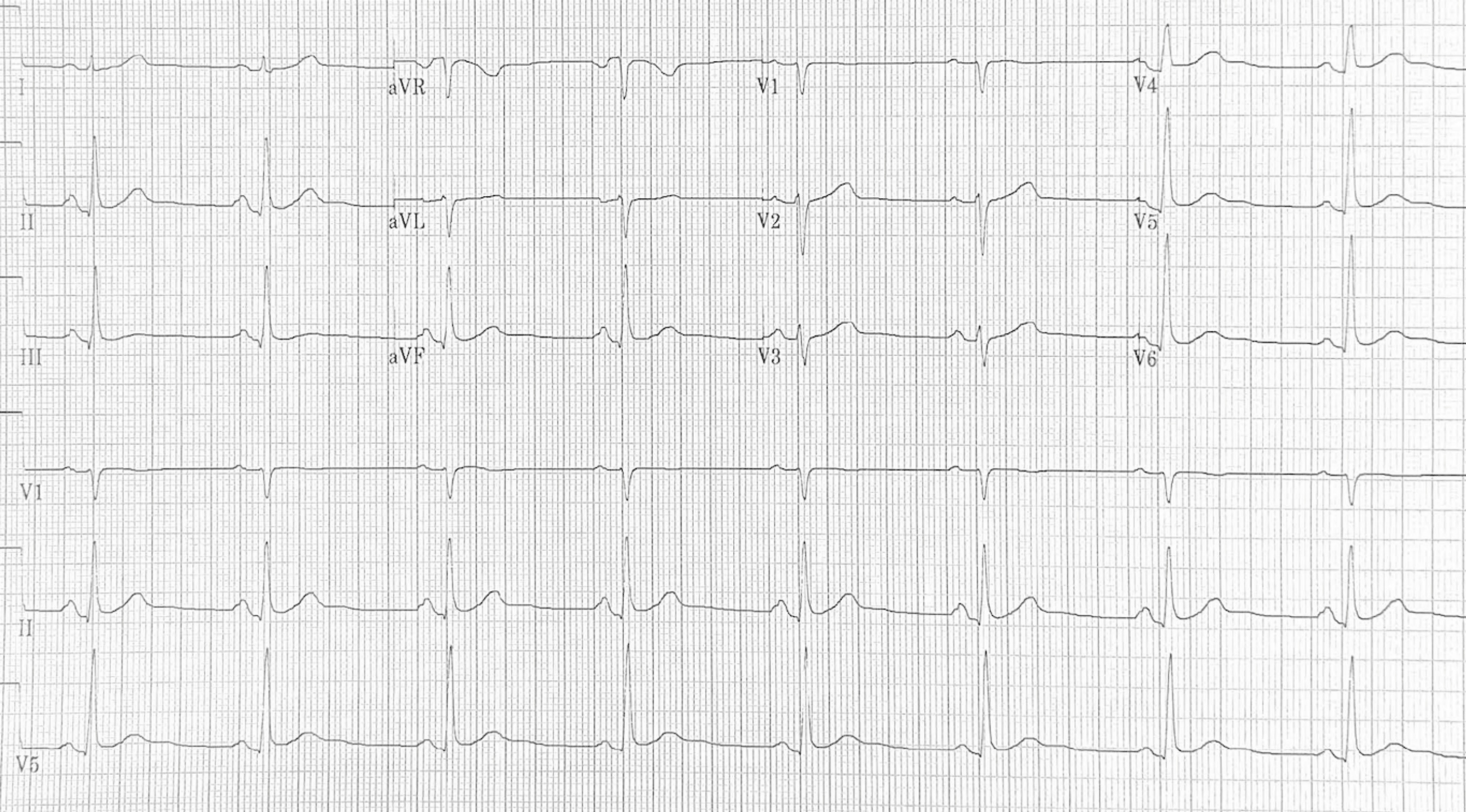
Baseline ECG before dronedarone initiation showing sinus bradycardia at 49 beats/minute and normal PR, QRS and QTc (410 ms) intervals.

Because the patient declined catheter ablation, rhythm-control therapy with dronedarone 400 mg twice daily was initiated. An ECG obtained on the day dronedarone was started showed sinus bradycardia at 49 beats/minute with normal PR, QRS, and QTc intervals (410 ms) (Figure 1). This lower heart rate was not present on his pre-treatment atrial fibrillation tracings and was attributed to an early effect of dronedarone rather than intrinsic sinus node dysfunction. Given the absence of conduction disease and the patient’s preference for a non-invasive rhythm-control strategy, a monitored trial of dronedarone was considered appropriate.

**Figure 2:**
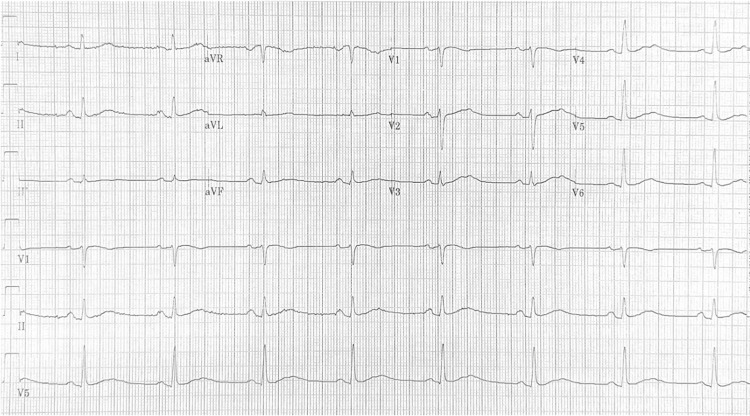
Follow-up ECG one week after starting dronedarone showing marked QTc prolongation at 563 ms.

After one week of dronedarone therapy, a repeat ECG (Figure 2) demonstrated marked QTc prolongation to 563 ms by Bazett’s correction. The patient remained completely asymptomatic, with no ventricular ectopy, pauses, or episodes of torsade de pointes. Dronedarone was promptly discontinued, and follow-up ECGs within one week showed normalization of the QTc interval to baseline values.

He was subsequently transitioned to flecainide 100 mg every 12 hours, which he continues to tolerate well without QT prolongation or further arrhythmic events.

## Discussion

Dronedarone is a multichannel antiarrhythmic agent with properties spanning Vaughan-Williams classes I-IV. It was developed as a non-iodinated analogue of amiodarone to preserve antiarrhythmic efficacy while minimizing long-term organ toxicity [1,2]. Its electrophysiologic actions include blockade of sodium channels (Class I), β-adrenergic receptors (Class II), potassium currents, including IKr (Class III), and calcium channels (Class IV) [3]. These effects contribute to both rhythm and rate control, but despite the initial appeal, there is accumulating clinical evidence and post-market surveillance that also raise concern for proarrhythmia, particularly via QT interval prolongation.

In large clinical trials [2], mean QTc prolongation with dronedarone has generally been modest, in the range of 10-25 ms, with maximal effects typically seen within three to seven days of initiation and resolution upon discontinuation. The ATHENA trial demonstrated an average increase of 10-20 ms, and torsade de pointes were rare. FDA labeling reports a mean increase of approximately 15 ms, with QTc >500 ms observed in ~1.7% of treated patients [4]. However, post-marketing data and case reports have described extreme QT prolongation (>700 ms) [5] and torsade, even in patients without overt risk factors.

The present case is notable because the patient had no structural heart disease or conventional risk factors for acquired long QT syndrome, yet developed marked QTc prolongation (563 ms) after only one week of therapy, justifying therapy discontinuation. Importantly, this occurred in the absence of symptoms, premature ventricular contractions, or other arrhythmic markers, underscoring that significant drug-induced repolarization abnormalities can remain clinically silent. Mild untreated obstructive sleep apnea may have contributed to the patient’s atrial fibrillation recurrence, but it is not known to increase susceptibility to drug-induced QT prolongation and is therefore unlikely to have influenced the repolarization abnormality observed [6,7].

Mechanistically, dronedarone prolongs ventricular repolarization via IKr blockade, predisposing to early afterdepolarizations and torsade de pointes. Unlike amiodarone, which appears to reduce transmural dispersion of repolarization, dronedarone may increase it, thereby enhancing arrhythmic susceptibility under certain conditions. Analyses of the FDA Adverse Event Reporting System [4] between 2009 and 2011 support these concerns, showing more reports of torsade de pointes, ventricular arrhythmia, and cardiac arrest with dronedarone compared with amiodarone, despite the latter’s much higher use.

The PALLAS trial, which enrolled patients with permanent atrial fibrillation and structural heart disease, was terminated early due to excess cardiovascular and arrhythmic deaths in the dronedarone arm [8]. While the ATHENA trial suggested cardiovascular benefit in lower-risk patients with paroxysmal atrial fibrillation [9], our case illustrates that even this population may experience clinically relevant proarrhythmia.

Although the dronedarone prescribing information lists baseline sinus bradycardia <50 beats/minute as a contraindication, the patient’s bradycardia developed after initiation of therapy and was not present beforehand. Thus, in this case, it represented an early drug effect rather than a baseline contraindication. The patient was also taking pantoprazole, which has minimal cytochrome P450 inhibition and is considered the proton pump inhibitor with the lowest potential for drug-drug interactions [10], making it unlikely to have influenced dronedarone metabolism or contributed to QT prolongation. This distinction is clinically relevant when interpreting the sequence of events and the patient’s overall arrhythmic risk profile.

Using the World Health Organization-Uppsala Monitoring Centre causality assessment system, the relationship between dronedarone and the QTc prolongation in this case meets the criteria for a probable adverse drug reaction [11]. The clear temporal association, rapid normalization of the QTc interval following drug withdrawal, absence of alternative explanations, and a well-established electrophysiologic mechanism collectively support dronedarone as the causative agent.

Although this patient did not develop torsade de pointes or other ventricular arrhythmias, QTc values above 500 ms are associated with increased proarrhythmic risk. Taken together, this case highlights that dronedarone, despite its reputation as a safer alternative to amiodarone, can produce substantial electrophysiologic disturbances, including marked QTc prolongation, in otherwise low-risk individuals. Prompt recognition of QT prolongation and immediate discontinuation remain critical to reducing the likelihood of progression to malignant arrhythmias such as torsade de pointes.

## Conclusions

Although dronedarone was designed as a safer alternative to amiodarone, this case highlights that it can still produce substantial disturbances in ventricular repolarization, including marked QTc prolongation above 500 ms, even in a young patient without structural heart disease or conventional risk factors. The clinically silent nature of the QTc abnormality in our patient underscores the limitations of symptom-guided follow-up and the importance of systematic ECG monitoring during therapy initiation and early follow-up. By documenting significant, asymptomatic QTc prolongation in an otherwise low-risk individual, this case contributes to the evolving understanding of dronedarone’s safety profile and underscores the need for vigilance in routine practice. Early recognition of QTc prolongation and prompt discontinuation of therapy remain essential to reducing the risk of progression to malignant arrhythmias such as torsade de pointes.
